# Genetic characterization of parvoviruses circulating in turkey and chicken flocks in Poland

**DOI:** 10.1007/s00705-012-1446-0

**Published:** 2012-08-17

**Authors:** Katarzyna Domanska-Blicharz, Anna Jacukowicz, Anna Lisowska, Zenon Minta

**Affiliations:** Department of Poultry Diseases, National Veterinary Research Institute, Al. Partyzantow 57, 24-100 Pulawy, Poland

**Keywords:** Parvovirus, Detection, Turkey, Chicken, Phylogenetic analysis, Recombination

## Abstract

Between 2008 and 2011, commercial turkey and chicken flocks in Poland were examined for the presence of turkey parvovirus (TuPV) and chicken parvovirus (ChPV). Clinical samples (10 individual faecal swabs/flock) from 197 turkey flocks (turkeys aged 1 to 19 weeks) and 45 chicken flocks (chickens aged 3 to 17 weeks) were collected in different regions of the country and tested using a PCR assay that targeted the NS1 gene (3’ORF). The prevalence of TuPV was 29.4 % in the flocks tested, while ChPV infections were found in 22.2 % of the studied flocks. Phylogenetic analysis revealed a clear division into three groups: ChPV-like, TuPV-like and a third, previously unrecognized and distinct subgroup, TuPV-LUB, containing exclusively three Polish isolates from turkeys. The isolates from the novel group showed as little as 50.6-64.5 % of nucleotide sequence identity to the prototype chicken and turkey parvovirus strains. Genetic analysis of a ChPV isolate that was classified in the TuPV group strongly suggests a recombination event between chicken and turkey parvoviruses.

Parvoviruses are small, icosahedral, non-enveloped particles, typically 20 nm in diameter [18]. Their genome is a linear, single-stranded DNA that is about 5 kilobases long and contains at least three open reading frames (5’ORF1, 3’ORF1 and a small ORF located between the major 5’ and 3’ ORFs). 5’ORF encodes a non-structural protein NS1, and 3’ORF probably encodes the capsid proteins VP1, VP2 and VP3. The role of a small ORF remains unknown [3].

Viruses belonging to the family *Parvoviridae* are much diverged and are classified into two subfamilies (*Densovirinae* and *Parvovirinae*), which are further classified in nine genera (4 within *Densovirinae* and 5 within *Parvovirinae*). These viruses infect a wide spectrum of hosts, ranging from insects to primates, and have different molecular characteristics that reflect their various biological features including tissue or host tropism [6]. The diseases caused by parvoviruses that are most familiar to aviopathologists are Derzsy’s disease in young geese and the syndrome known as MMDR (from French “mortalité, morbidité, deplument, reptation”) in Muscovy ducks [4]. These parvoviruses belong to the genus *Dependovirus*. A distinct group in the subfamily *Parvovirinae* has recently been recognized as the possible causative agents of enteric and sometimes also neurologic disease symptoms in turkeys and chickens [4, 11]. The studies revealed that their genome is slightly different and that they cluster into separate, usually host-specific groups, namely chicken parvovirus (ChPV) and turkey parvovirus (TuPV) [3, 20].

The major enteric disease complex in turkeys characterized by diarrhea, thermoregulatory disorders, depression, growth retardation and increased feed consumption is called poult enteritis complex (PEC), and in its more severe form, with acute mortality, stunting and thymic lesions, it is known as poult enteritis mortality syndrome (PEMS). In chickens, two terms are used alternately for description of the enteric disease complex: runting-stunting syndrome (RSS) and maladsorption syndrome (MAS) [1, 5]. The causes of these diseases are complex and polymicrobial. However, reports that, among other factors, parvoviruses could also be responsible for enteric diseases in turkeys and chickens originate from the mid-80s [7, 8, 19].

The aim of the present study was to investigate the prevalence of parvovirus infections in commercial meat-type turkey and chicken flocks in Poland and to estimate their genetic relatedness.

Between January 2008 and October 2011, a total of 1970 faecal swabs were collected from 197 turkey flocks (10 individual faecal swabs/flock) located in different regions of Poland. Samples were collected from turkeys aged 1 to 19 weeks. Most of the flocks tested had individual birds that showed one or more PEC or PEMS symptoms, but in other flocks, the birds were in good health. Beginning from May 2009, samples from chickens were also collected. Faecal swabs and different organ/tissue samples were obtained. They originated from 45 commercial chicken flocks at different ages (3-17 weeks old) that were clinically healthy or had RSS. All samples were stored below −20 °C until processing. After slow thawing, each individual swab was hydrated in phosphate-buffered saline (PBS) containing 2,000 U/ml of penicillin and 2 mg/ml of streptomycin, incubated for 1 h at room temperature, and clarified by centrifugation at 1,500g for 20 min. Tissue samples were homogenized in the same PBS with antibiotics (10 % w/v), incubated at room temperature for 20 min and clarified by centrifugation at 3,000×*g* for 15 min.

DNA was extracted from 250 μl of supernatant from five pooled swabs (2 pools/flock) and from tissue homogenates using a DNeasy Mini Kit (QIAGEN, Germany) according to the manufacturer’s instructions. Each DNA was eluted in 50 μl molecular-grade water. PCR assay directed toward the NS1 gene (3’ORF) was used for parvovirus detection [21]. The products were separated on a 2 % agarose gel in Tris-acetate-EDTA buffer and visualized by ethidium bromide staining. The 561-bp amplicons obtained by PCR from 24 positive flocks (18 turkey and 6 chicken flocks) were sequenced in both directions by Genomed Sp. z o.o. (Warsaw, Poland). Each sequencing reaction was repeated three times for atypical TuPV strains and once for the remaining isolates. Using the SeqMan program (DNASTAR, Madison, WI), the forward and reverse nt sequences were aligned as one consensus sequence. Multiple alignments of nt and aa sequences were performed using the MegAlign application (DNASTAR, Madison, WI) using the Clustal W method. Phylogenetic analysis of aligned sequences was performed with MEGA 5.0 using the neighbor-joining method with the maximum-likelihood model. Bootstrap scores were generated from 1000 replicates. The nt sequences were translated to putative amino acid (aa) sequences, which were also compared to detect any changes at the aa level. Selected Polish sequences were screened for possible recombination using different programs available in the RDP4 software package with their default parameters [10]. Sequences determined in this study have been submitted to GenBank with accession numbers JQ178299-JQ178322.

Of the 197 turkey flocks tested, 58 (29.4 %) birds were positive for parvovirus infection. Among parvovirus-positive flocks PEC (most flocks), PEMS (7 flocks) and no enteric symptoms (3 flocks) were observed. The age of positive turkey flocks ranged from 1 to 19 weeks-old, with the majority 3-7 weeks old. Chickens were positive for parvovirus infection in 10 (22.2 %) of the flocks tested. The presence of a parvoviral genome in swabs but also in different organs, including intestines, kidneys, trachea and pancreas, was found in healthy as well as in chickens suffering from RSS symptoms.

Polish avian parvovirus isolates are divided into three groups: ChPV-like, TuPV-like, and a third, separate, and previously unrecognized group containing exclusively three turkey parvovirus strains (designated as TuPV-LUB; LUB is the abbreviation for the village where isolates were detected) (Fig. 1). A comparison of nucleotide (nt) and amino acid (aa) sequences (from nt 1474 to 1981 of the prototype TuPV 1078 strain) of 14 Polish turkey parvovirus strains from group TuPV showed 97.4-100 % (nt) and 95.9-100 % (aa) similarity to each other and 98.0-99.6 (nt) and 96.4-100 % (aa) similarity to the prototype TuPV 1078. Among isolates from the ChPV-like group; the nt sequence identity was 97.8-99.0 %, and the deduced amino acid sequence identity was 97.0-100 %. In turn, the nucleotide and amino acid similarity of these isolates to the prototype ChPV ABU P1 was 96.2-97.2 % and 97.0-97.6 %, respectively (from nt 1920 to 2384 of the ChPV ABU P1 genome). The phylogenetic position of the TuPV-LUB group was supported by a bootstrap value of 100 % at the main node. Comparing the 524-bp-long fragment of the NS gene of three atypical Polish TuPV-LUB isolates, the nucleotide sequence identity was 99.2-99.8 %, and the amino acid sequence identity was 97.7-99.4 %. However, they were distantly related to the prototype TuPV 1078 strain (from nt 1450 to 1989), with identities of 64.1-64.5 % at the nucleotide level and 50.6-51.1 % at the amino acid level, and also to the prototype ChPV ABU P1 strain (from nt 1860 to 2396), with identities of 63.7-63.9 % at the nucleotide level and 51.7-52.3 % at the amino acid level. The strain ChPV/Poland/G090/2011 [JQ178302], isolated from chickens, clustered in the TuPV-like group. The nt and aa sequences of its NS1 gene fragment were very similar to those of a recently described ChPV/Hun/1515/2007 strain that also clusters in the TuPV-like group (nt and aa identity, 98.4 and 98.2 %, respectively) and TuPV strain 1078 (nt and aa identity, 98.0 and 96.4 %, respectively). The possibility of recombination with a potential crossover site at the end of analysed NS1 gene fragment was suggested by three recombination detection methods implemented in the RDP4.13 software (GENECONV, MaxChi and Chimaera). A GENECONV plot revealed higher similarity and a phylogenetic relationship of TuPV-like ChPV/Poland/G090/2011 to TuPV/Poland/G193-K3/2008 in a small, about 30-nt fragment at the end of the NS1 region that was analysed (positions between 2320 and 2350 according to the full-length genome sequence of the reference ABU P1 strain), while the remainder of the analysed fragment had greater similarity to ChPV/Poland/G097/2011 (Fig. 2). It is therefore probable that the TuPV-like ChPV isolates arose from ChPV through the acquisition of a gene fragment from an atypical TuPV. The relatively low average P-values obtained by implementing recombination detection methods (1.53 × 10^−4^, 7.85 × 10^−3^, 4.6 × 10^−3^ in GENECONV, MaxChi and Chimaera, respectively) could result from the short region of NS1 gene analysed.

**Fig. 1:**
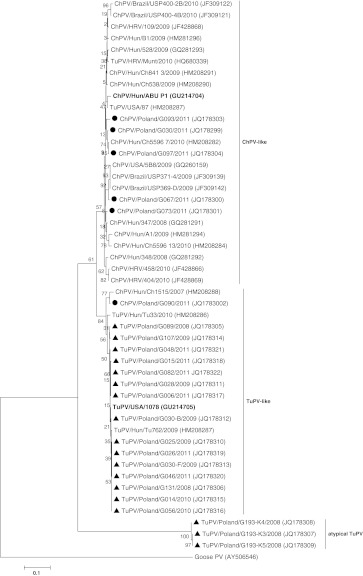
Phylogenetic tree of nucleotide sequences of the NS1 gene fragment of the Polish ChPV (indicated by a black dot) and TuPV strains from the present study (indicated by a black triangle), and sequences from GenBank. Sequences are identified by parvovirus host/country/code/year (accession no). Names of sequences in bold are strains that were used as references. Goose parvovirus strain HG5/82 from China was used as the outgroup. The phylogenetic tree was constructed using the neighbor-joining algorithm and the maximum-likelihood model with 1000 bootstrap replicates (bootstrap values shown on tree)

**Fig. 2:**
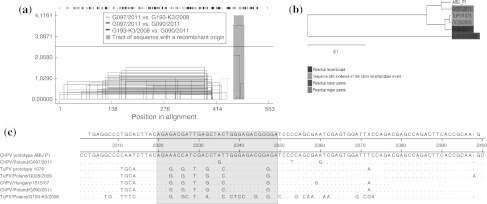
**a** Recombination analysis of the 540-nt fragment of the NS1 gene of selected turkey and chicken parvovirus strains by the GENCONV method. The region suspected to have arisen through recombination is indicated by pink shading. **b** The relationships between studied strains in this region. **c** Comparison of nt sequences around the recombination region (gray shading) of prototype strains ABU P1 and TuPV/1078 with selected Polish ChPV and TuPV

In the territory of Poland, birds infected with parvovirus were detected in 29.4 % of commercial turkey farms and in 22.2 % of commercial chicken farms. Slightly lower rates of parvovirus infection in Polish chicken farms were demonstrated previously [17]. However, the occurrence of ChPV and TuPV in Poland was lower when compared to the reported 77-78 % prevalence in commercial chicken and turkey flocks in a survey in the USA between 2003 and 2008 [21]. The presence of parvovirus infections in Hungarian and Croatian commercial chicken and turkey flocks was also reported recently, but their prevalence in those countries was not estimated [2, 14]. Turkey parvovirus was detected in 1- to 19-week-old (mostly 3- to 7-week-old) turkeys. The presence of TuPV in poults at 1 week of age is not surprising, as different authors have suggested the possibility of vertical transmission of the virus [8, 9]. In our studies, parvoviruses were mainly detected in flocks with enteric disease, but in a few parvovirus-positive flocks, chickens and turkeys did not show any symptoms of disease. This is in accordance with previous findings of Zsak et al., even though in another study, the presence of parvoviruses was not detected in poultry with no enteric disorders [13, 14, 21]. We also demonstrated the presence of parvovirus in the intestines, kidneys, ceacal tonsils, trachea and pancreas of 6-week-old broiler chickens. This may be the effect of infection with parvovirus strains differing in their pathogenicity. Recently, parvovirus-associated cerebellar hypoplasia, hydrocephalus and enteritis were diagnosed in 1-day-old broiler chickens, and the genome of ChPV was detected in the brains of affected chickens [11]. The nucleotide sequence of this strain [ChPV/USA/5B8/2009] as well as the sequences of Polish ChPVs detected in this study are very similar to those of other parvoviruses isolated from RSS cases, so it is possible that other regions of the genome are involved in pathogenicity. Also, complicating factors such as secondary bacterial, fungal or viral infections may exacerbate the course of parvovirus infections.

Genetic variability in the NS1 gene was observed among parvoviruses; two major groups (ChPV and TuPV) were described previously, but we have also found a third distinct group (TuPV-LUB) containing three atypical parvovirus strains isolated from turkeys. Five Polish ChPV isolates clustered closely with North American, Hungarian, Croatian and Brazilian strains isolated from chickens, whereas fifteen parvoviruses isolated from turkeys were assigned to the TuPV group together with strains from the USA and Hungary. The novel, formerly unrecognized group of parvoviruses designated TuPV-LUB was supported by very low nucleotide sequence similarity to the prototype TuPV strain (62.6-63.1 %) and the topology of the phylogenetic tree. The samples infected with atypical TuPV were collected from the same turkey farm at the same time. The birds originated from the same breeder flock but were kept in different age-group houses of 13,000 poults each: 2 weeks old (TuPV/Poland/G193-K3/2008 and G193-K5) and 4 weeks old (G193-K4). In all houses, the birds exhibited uneven growth, diarrhoea, and decreased water and feed consumption. In our study, we also found the isolate ChPV/Poland/G090/2011, which was assigned (together with a closely related Hungarian isolate) to the TuPV-like group. The uniqueness of this isolate was most related to the acquisition of the genome fragment as a result of a recombination between ChPV and an atypical TuPV. Until now, no recombination events have been identified in the genomes of TuPV and ChPV, but the finding of the new atypical TuPV-LUB group strongly suggests such a possibility, and it therefore seems probable that the pool of unknown parvoviruses circulating among poultry plays a role in virus evolution. Recombination possibilities within and among other parvoviral species were indicated recently [16]. The genetic diversity of the analysed NS1 fragment of Polish strains detected in this study may result from the way the virus replicates in host cells. Shackelton at al. indicated several mechanisms that were most likely to be responsible for the high mutation rate of single-stranded canine parvoviral DNA, including the involvement of a subset of the cellular machinery in parvoviral genome replication that changes the efficiency or accuracy of the polymerase, disturbance of proofreading and repair mechanisms or using rolling hairpin structures instead of the typical replication fork of the double helix needed for replication [15]. Such imperfect virus replication and the possibility of coinfection of host cells with different parvovirus strains would provide excellent conditions for the occurrence of recombination events [12, 14]. We found two turkey strains (TuPV/Poland/G030-B/2009 and G030-F/2009; JQ178312 and JQ178313, respectively) in the same flock sampled at the same time, and they differed in one nucleotide, resulting in an aa change (a missense point mutation at position 1532 according to the full-length genome sequence of the reference strain TuPV 1078). This finding could be the result of coinfection with different strains or a mutation in a single viral strain.

In conclusion, the present study indicates the circulation of genetically diverse populations of TuPV and ChPV in Polish turkey and chicken flocks. The atypical TuPV-LUB group of turkey parvoviruses newly recognized in this study probably represent recombinant viruses coming into existence. However, in order to determine the probability of the occurrence of recombinant viruses, an effort should be made to sequence the whole genome of these strains.
